# Retention of Donor T Cells in Lymphohematopoietic Tissue and Augmentation of Tissue PD-L1 Protection for Prevention of GVHD While Preserving GVL Activity

**DOI:** 10.3389/fimmu.2022.907673

**Published:** 2022-05-23

**Authors:** Qingxiao Song, Ubaydah Nasri, Ryotaro Nakamura, Paul J. Martin, Defu Zeng

**Affiliations:** ^1^ Arthur D. Riggs Diabetes and Metabolism Research Institute, The Beckman Research Institute, City of Hope National Medical Center, Duarte, CA, Unites States; ^2^ Hematologic Malignancies and Stem Cell Transplantation Institute, City of Hope National Medical Center, Duarte, CA, Unites States; ^3^ Fujian Medical University Center of Translational Hematology, Fujian Institute of Hematology, and Fujian Medical University Union Hospital, Fuzhou, China; ^4^ Fred Hutchinson Cancer Research Center, University of Washington, Seattle, WA, United States

**Keywords:** graft versus leukaemia (GVL), graft versus host disease (GVHD), naive T cell depletion, PD-L1, chemokine and chemokine receptors, anti-IL-2

## Abstract

Allogeneic hematopoietic cell transplantation (Allo-HCT) is a curative therapy for hematological malignancies (i.e., leukemia and lymphoma) due to the graft-versus-leukemia (GVL) activity mediated by alloreactive T cells that can eliminate residual malignant cells and prevent relapse. However, the same alloreactive T cells can cause a serious side effect, known as graft-versus-host disease (GVHD). GVHD and GVL occur in distinct organ and tissues, with GVHD occurring in target organs (e.g., the gut, liver, lung, skin, etc.) and GVL in lympho-hematopoietic tissues where hematological cancer cells primarily reside. Currently used immunosuppressive drugs for the treatment of GVHD inhibit donor T cell activation and expansion, resulting in a decrease in both GVHD and GVL activity that is associated with cancer relapse. To prevent GVHD, it is important to allow full activation and expansion of alloreactive T cells in the lympho-hematopoietic tissues, as well as prevent donor T cells from migrating into the GVHD target tissues, and tolerize infiltrating T cells *via* protective mechanisms, such as PD-L1 interacting with PD-1, in the target tissues. In this review, we will summarize major approaches that prevent donor T cell migration into GVHD target tissues and approaches that augment tolerization of the infiltrating T cells in the GVHD target tissues while preserving strong GVL activity in the lympho-hematopoietic tissues.

## Introduction

Allogeneic hematopoietic cell transplantation (Allo-HCT) provides curative therapy for hematological malignancies such as lymphoma and leukemia by relying on the graft-versus-leukemia/lymphoma (GVL) effects mediated by alloreactive T cells (1–6). However, the same alloreactive T cells also cause a severe side effect, graft-versus-host disease (GVHD). Prevention of GVHD while preserving GVL activity remains a long-sought and elusive goal.

Acute GVHD (aGVHD) is a dysregulated and exaggerated alloimmune response whose pathogenesis has been elegantly described in previous and recent reviews (7–11). Although GVHD and GVL activity are mediated by the same alloreactive T cells, they occur in different tissue compartments. GVHD target tissues include the skin, lung, liver and intestine (12), but leukemia/lymphoma cells reside mainly in lympho-hematopoietic tissues, including the bone marrow, spleen and lymph nodes (13). Current methods that suppress general alloreactive T cell activation and expansion such as immunosuppressants (i.e., tacrolimus and sirolimus) simultaneously reduce GVHD and GVL activity (14, 15). Blocking alloreactive T cell infiltration in GVHD target tissues while allowing full activation and expansion of alloreactive T cells that kill malignant cells in the lympho-hematopoietic compartment offers a better approach toward prevention of GVHD. In addition, cellular therapy such as infusion of Tr1 can prevent GVHD while exerting GVL activity in pre-clinical models (16–18) and can maintain alloreactive responses without causing GVHD in humans (19, 20). Moreover, CD8^+^ Tregs can enhance GVL activity while suppressing GVHD (21–23). In this review, we focus on the approaches that can maintain donor T cells in lymphohematopoietic tissue and augmentation of tissue PD-L1 mediating protection to prevent GVHD while preserving GVL activity. We summarize preclinical studies and clinical trials that have tested this compartmental approach for preventing GVHD while preserving GVL activity.

## Inhibition of Tissue-Specific T Cell Homing and Chemokine Receptors

T cell migration into GVHD target tissues requires specific homing and chemokine receptor expression and release of the corresponding of chemokines in tissues. In general, T cell that express CXCR3, CCR9 or α_4_β_7_integrins interacting with their ligands CXCL9, CXCL10, CXCL11, CCL25 or MAdCAM-1 migrate into gut (24). T cell that express CCR5 or α_4_β_1_integrins interacting with its ligands CCL3, CCL4, CCL5 or VCAM-1 migrate into liver (24, 25). T cells that express CCR3, CCR4, or CCR6 interacting with their ligands CCL11, CCL17/CCL22 or CCL20 migrate into lung and skin (24, 26–28).

Various studies have tested the effects of targeting chemokine and chemokine receptors in experimental murine GVHD models. For example, a decrease in skin, liver and gut GVHD can be achieved through elimination of CXCR3^+^ T cells, or by neutralization of its ligands CXCL9-11 (29–31). Based on these results, several patent applications for CXCR3 antagonists have been filed, but no such antagonist has been approved for prevention of GVHD or treatment of other diseases in humans (32). Another novel approach involves the use of CXCR3-transfected regulatory T cells (Tregs) that migrate and accumulate in the liver, lung and gut, resulting in decreased GVHD severity (33). CCR5 is an important receptor that allows lymphocytes to migrate to the skin and causes the production of TNF-α, IL-2, and IFN-γ, leading to development of aGVHD in patients (34). A 32-nucleotide deletion of CCR5 (CCR5Δ32) in recipients significantly decreased the risk of GVHD (35). Moreover, in both recipient and donor cells, the CCR5Δ32 genotype exhibited the greatest protection (35). This protection may depend on the conditioning regimen (36). Maraviroc, which is an inhibitor of CCR5, has been approved for treatment of HIV infection. An initial report about Maraviroc for prevention of GVHD in human showed encouraging results (37), but a follow-up study did not (38).

CCR9 plays a critical role in the homing of lymphocytes into inflamed intestines, thereby contributing to the pathogenesis of colitis and Crohn’s disease (39). An orally bioactive inhibitor of CCR9, CCX282, has been developed and was well tolerated with encouraging results in clinical trials for Crohn’s disease (40), but no studies in treatment of GVHD has been reported so far. Surprisingly, in a murine GVHD model, lack of CCR9 expression by donor T cells did not ameliorate GVHD since CCR9 deficiency on donor T cells did not impact on inflammatory cytokine production and T cells accumulation in liver and intestine (41). These results suggested CCR9 seems to have a subordinate role for donor T cell homing *in vivo* during aGVHD.

The integrin α_4_β_7_ has a critical role in mediating aGVHD. Upregulation of α_4_β_7_ integrin expression by T cell subsets correlates with the development of gut aGVHD in humans (42, 43). In murine models, the severity of GVHD was lower with α_4_β_7_ deficient donor T cells than with wild-type (WT) T cells (41, 44). The α_4_β_7_ deficient donor T cells also showed intact graft-versus-tumor (GVT) activity or even enhanced activity (44). Similarly, MAdCAM-1 deficiency on recipients reduced GVHD in mice (41), moreover, administration of anti-MAdCAM-1 antibody reduced GVHD without impairing GVL effects in both nonirradiated recipients and in recipients treated with myeloablative conditioning (45). Recent study found intestinal stem cells were the primary target of alloreactive donor T cells (46). Furthermore, it was shown that this process relies on β7 integrin and MAdCAM-1 interactions, since the anti-MAdCAM-1 antibody reduced donor T cell invasion into the lower crypt regions of the mucosa resulting in less damage to the GI tract (46). Vedolizumab, a monoclonal antibody that binds to α_4_β_7_, has been approved for treatment of ulcerative colitis and Crohn’s disease and, more recently, has been examined as a treatment for steroid refractory gut GVHD with variable results (47–51), however there is no information about the use of Vedolizumab to prevent GVHD.

T cell tissue tropism and expression of chemokine receptors is imprinted by tissue CCR7^+^ dendric cells (DCs) in the draining lymph nodes (52–56). Consistently, anti-CD3-preconditioning effectively depletes CCR7^+^ DCs in mesenteric lymph nodes by inhibiting CCR7^+^ DC migration from gut tissue into MLN and inducing CCR7^+^ DC apoptosis in the MLN. Consequently, the expression of gut homing molecules α4β7 and CCR9, as well as skin homing molecules E-Lig, P-Lig, CCR4 and CCR10, is reduced in donor T cells. Additionally, anti-CD3 preconditioning reduced the expression of CCL17, CCL22, CCL27, and CCL28 in skin tissues. Anti-CD3 preconditioning also reduced gut, skin, and liver tissue expression of CCL3-5 and CXCL9-11, which decreased alloreactive donor T cell tissue tropism towards skin, liver, and gut. Therefore, anti-CD3 preconditioning allows fully activated donor T cells to be retained and mediate GVL activity in lympho-hematopoietic tissues where hematological cancer cells reside without causing GVHD (57, 58). Due to lack of depleting anti-human CD3 mAb, this approach has not yet been tested in humans.

Sequestering lymphocytes within secondary lymphoid organs offers an alternative to preventing GVHD by blocking migration of alloreactive donor lymphocytes to target tissues in the recipient. Exit of T cells from lymphoid tissues require their expression of sphingosine 1-phosphate receptors (S1PRs). The S1PRs agonist FTY720 retains alloreactive T cells in lymphoid tissues and prevents T cell infiltration of GVHD target tissues, thereby preventing GVHD while preserving GVL effects in both MHC mismatched and MHC-haploidentical murine GVHD models (59–62). On the other hand, FTY720 also reduced the numbers of host DCs in the recipient spleen before transplantation and slightly impaired GVL activity (63, 64). Fingolimod (FTY720), a first-in-class, orally bioavailable S1PR agonist has been approved in 2010 for treatment of relapsing forms of multiple sclerosis (MS). Published clinical testing for GVHD in humans, however, is limited to a single a 66-year-old patient with severe CNS GVHD who was treated successfully with fingolimod (65). Overall, few studies have evaluated pharmacological targeting of chemokines and chemokine receptors in clinical settings. This approach might be valid for treatment, but not for prevention of GVHD, if the inhibitor is given prior to the infusion of donor cells. In addition, the difficulty in controlling donor T cell migration may be promoted by pre-existing tissue resident T cells in the human GVHD target tissues (66).

## Reduction of Target Tissue Inflammation

Tissue inflammation caused by pretransplant conditioning regimens triggers migration of alloreactive T cell into GVHD target tissues (67). Delayed lymphocyte infusion (DLI) of donor T cells after tissue inflammation has subsided reduced GVHD while augmenting GVL effect in murine models and humans (68–75). The DLI mediated GVL effect has been confirmed for chronic myeloid leukemia (CML) in numerous studies worldwide (76, 77), with up to 70–80% cytogenetic complete remissions (78). For patients with acute myeloid leukemia (AML) or myelodysplastic syndrome (MDS), the response rate to DLI is much lower (20–40%) and is lower still in those with acute lymphoid leukemia (ALL) (10–13%) (79).

According to most studies, conditioning regimens initiate aGVHD by triggering the production of cytokines (such as TNF-α, IFN-γ, IL-1, and IL-2) which, in turn, up-regulate the chemokine receptors and their ligands that drive the migration of T cells to GVHD target tissues (53, 55, 80, 81). Certain cytokines promote GVHD while also providing survival signals to leukemia cells. These include granulocyte-macrophage colony-stimulating factor (GM-CSF) (82, 83) and macrophage colony-stimulating factor 1 (CSF-1) (84) in AML, and Interleukin-6 (IL-6) in ALL (85) and multiple myeloma (MM) (86). Among these, the role of IL-6 has been demonstrated in the pathogenesis of GVHD in several murine GVHD models (87, 88). Expression of IL-6 and IL-6R is enhanced after allo-HCT, DCs are the principal source of IL-6 dysregulation after allo-HCT, and blockade of IL-6 signaling by *in vivo* administration of anti-IL-6R mAb attenuates GVHD with significant expansion of Tregs and reduction of inflammatory Th1 and Th17 cells (87, 88). Inhibition of classical signaling of IL-6R on donor T cells decreased the severity of Th17 and Th22-dependent GVHD without inhibiting GVL response against a primary blast crisis chronic myeloid leukemia cell line BCR-ABL/NUP98-HOXA9 (88). Similarly, tumor necrosis factor-alpha (TNF-α) blockade ameliorated GVHD mediated by both CD4^+^ and CD8^+^ T cells without blocking GVL activity (89). Another study, however, showed that recipients given TNF-α receptor deficient T cells had a significant impairment in donor GVL activity after HCT compared to recipients of WT T cells, indicating that TNF-α has an important role in GVL mediated by donor T cells (90). Neutralization of a single cytokine such as IL-6 and TNF has shown variable and conflicting results (89, 91, 92). A recent study, however, showed beneficial effects from dual blockade of both IL-6 and TNF in prevention of GVHD in both MHC-mismatched and minor antigen-mismatched aGVHD murine models and in sclerodermatous cGVHD murine models (93), while preserving GVL activity against A20 (B-cell lymphoma) and C1498 (acute myeloid leukemia) (93). In clinical trials, however, tocilizumab (TCZ), a monoclonal antibody against the interleukin-6 receptor, did not significantly reduce the incidence of grade 2-4 aGVHD, and did not improve long-term survival (94). Similarly, the clinical results of testing TNF blockade for prevention of GVHD have been disappointing (95, 96).

## Infusion of Mesenchymal Stem Cells (MSCs)

MSCs are highly heterogeneous population of stem and progenitor cells that can be isolated and expanded from many tissues, such as bone marrow, placenta, umbilical cord (UC), adipose tissue (AT), and dental pulp (97–102). It has been demonstrated that MSC heterogeneity occurs within the same species, the same tissue preparations, and even on the same donor isolations (103–106). In general, MSCs inhibit the generation of cytotoxic T cells by secreting a soluble factor, but they do not interfere with CTL and NK cell lytic activity (107). Other studies have suggested that tolerance induction by MSCs may occur through inhibition of dendritic cell maturation and function (108–110), induction of myeloid-derived suppressor cells (MDSCs) (111), and suppression of B cells (112). Although it has been difficult to recover MSCs from BM of transplant recipients, MSCs can migrate to lymphoid organs and engraft at areas of tissue damage or tumor progression (113–115). These results indicate that MSCs are recruited mainly to tissues other than bone marrow for immune suppression in GVHD. The ability of MSCs to suppress infiltration of activated T cells into GVHD target tissues but not into the bone marrow contributes to the separation of GVL from GVHD. In addition, MSCs ameliorate GVHD through expansion of Tregs, especially the CD8^+^ Tregs (116–118). Unlike CD4^+^ Tregs, CD8^+^ Tregs suppress GVHD while preserving GVL activity (21–23). In clinic, third party, ex-vivo expanded, MSCs co-injection in a high risk, mismatched, unrelated-donor HCT reduced the severity of GVHD (119). Co-injection of MSCs and HSCs in HCT with HLA-identical sibling donors reduced the severity of aGVHD, but the incidence of relapse was significantly increased (120), and a comprehensive meta-analysis showed that co-administration of MSCs with allo-HCT has no demonstrable benefit regarding engraftment or prevention of aGVHD or cGVHD (121).

Exosomes are naturally occurring extracellular vesicles (EVs) that are released from many cell types and can be enriched from virtually all body fluids, including blood plasma, urine and saliva (122). Depending on their origin, some exosomes exert immune stimulatory or immune suppressive functions (122, 123). Since MSC exosomes represent a therapeutically active product of MSCs, it was suggested that EVs could have similar tissue repair capabilities as MSCs, making them a promising noncellular approach for GVHD prevention or treatment (124). In murine models bone marrow MSC derived EVs enhanced survival and reduced the severity of aGVHD (125), but MSC-EVs have not been tested for prevention of GVHD in humans.

Overall, the efficacy of MSCs treatment varies from study to study, possibly because MSCs are very heterogeneous, depending on their origin and the methods used to isolate and propagate them *in vitro*. Progress will require improved understanding of the mechanisms of MSCs and the development of methods that define the optimal source, *in vitro* culture methods, measurement of potency, cell dose, and the timing and frequency of administration.

## Depletion of Naïve Donor T Cells Reduce Tissue Infiltration and Reduce GVHD While Preserving GVL Effect

Generally, T cells can be divided into two types: (1) naïve T cells (T_N_) which have not yet encountered their corresponding antigens, and (2) antigen-experienced T cells, which include memory and effector T cells (T_M_) composed primarily of clonal expansions of T cells specific to their respective antigens (126). Based on the phenotype, gene expression, metabolic profile, and function of these antigen-experienced T cells, they can further be subdivided into three main types: central memory T cells (T_CM_), effector memory T cells (T_EM_), and effector T cells (T_E_) (127). Most T cells in the blood of mice have a T_N_ phenotype and both CD4^+^ and CD8^+^ T cells from peripheral blood mediated lethal GVHD in an MHC-mismatched HCT model (128). In contrast, bone marrow T cells are T_M_ phenotype that failed to induce lethal GVHD but retained GVL activity and facilitated hematopoietic progenitor engraftment (128). This study indicated that preserving resident marrow T_M_ cells but not blood T_N_ cells in the transplant inoculum may result in the desirable outcome of GVL and facilitation of engraftment without causing GVHD.

In subsequent studies, several different groups evaluated T_N_ and T_M_ subsets for their ability to cause GVHD using various murine GVHD models (129–137). The models involved in these studies included different MHC disparity (e.g., MHC-mismatched, MHC-matched, and minor H antigen mismatched), distinct GVHD disease patterns (aGVHD and cGVHD), as well as CD8^+^ and CD4^+^ T cell-mediated models respectively. Consistently, T_N_ caused severe GVHD, while T_EM_ did not cause GVHD. Some studies showed that T_CM_ can also cause intestinal damage that was less severe than with T_N_ (131). More importantly, the CD8^+^ T_M_ preserve GVL function *in vivo* (133, 135). Taken together, these preclinical murine studies indicated that T_N_ consistently mediate GVHD, while T_M_ either do not cause GVHD or cause only mild GVHD while still contributing to functional GVL effects.

Preclinical studies showed that human T_N_ and T_M_ have distinctly different fates after alloactivation *in vitro* (138). T_M_ lost their function to recognize alloantigens, whereas the T_N_ remained highly functional (138). These results suggested that T_N_-depletion was likely to reduce the expansion of alloreactive T cells after allo-HCT. Based on these discoveries, a single-arm clinical trial was designed to evaluate outcomes after CD45RA^+^ T_N_-depleted allo-HCT. Accordingly, 35 patients with high-risk leukemia received T_N_-depleted peripheral blood stem cell transplantation (PBSCT) from HLA-matched sibling donors after myeloablative conditioning (139). During the first three months after HCT, T cell immune reconstitution was comparable to that with unmanipulated bone marrow transplant (BMT) and was significantly better than with CD34^+^ selected pan-T cell depleted (TCD) PBSCT recipients, although the incidence of moderate (grade II-III) aGVHD was not reduced. GVHD in these patients, however, was uniformly responsive to corticosteroids, with a very low incidence of grade III-IV GVHD. The incidence of cGVHD was strikingly reduced compared to BMT. More importantly, the presence of T_M_ in the graft contributed to rapid recovery of T cells and the transfer of protective virus-specific immunity. No excessive rates of infection or relapse was observed (139). Similar observation was reported recently on three prospective phase II clinical trials of 138 patients with acute leukemia and MDS received T_N_-depleted PBSCT from HLA matched related or unrelated donors, aGVHD was mild and corticosteroid-responsive; Strikingly, only 7% of patients developed cGVHD, which was also mostly mild and steroid-responsive. No apparent increase in relapse or fatal infections (140). T_N_-depletion of PBSCT is also being applied to the setting of HLA-haploidentical HCT (haplo HCT) (141–144). In a recent report summarizing preliminary data from the first 50 subjects enrolled in an ongoing clinical trial, the results indicated an increase in the 3-year overall survival (OS) and event-free survival (EFS) in non-chemo refractory recipients receiving T_N_-depleted grafts (78.9% and 77.7%, respectively) compared to historic T-cell depleted haplo HCT cohorts (46.7% and 42.7%, respectively; *p* = 0.004 and 0.003 respectively) (144). Based on these results, clinical trials are now in progress to compare T_N_-depleted PBSCT with standard unmanipulated allo-HCT along with other promising GVHD-reduction strategies (145, 146).

Collectively, the outcomes of T_N_-depleted allo-HCT are very encouraging, and the understanding obtained from various human studies generally correlate with the results of murine experiments. However, relapses still occur at a low incidence. The underlying mechanism by which T_N_ and T_M_ exert differential effects on alloreactivity remains unclear. It has been proposed that T_N_ cause GVHD while T_EM_ do not because they lack CD62L and CCR7, which are critical in directing T_N_ toward to the sites of antigen presentation for GVHD initiation, such as lymph nodes (LN) and Peyer patches (PP). However, in murine GVHD model, Anderson et al. showed that CD62L and CCR7 were not required for T_N_-mediated GVHD, since CD62L^-/-^ donor T cells still induced GVHD, and GVHD also developed in recipients that lacked LN and PP. Even when T_EM_ constitutively express CD62L, they do not cause GVHD (147), indicating that targeting a single chemokine receptor alone on T_N_ cells might not be an effective therapy. Therefore, to further clarify the mechanism in human, future studies are needed evaluate the difference between T_N_ and T_M_ related to the respective cell trafficking patterns, or whether pathogen-specific T_M_ cross react with leukemia/lymphoma-associated antigens but with little cross-reactivity for alloantigen will need further investigation.

## Host Tissue PD-L1 With Donor CD4^+^ and CD8^+^ T Cells

PD-L1 interact with receptors PD-1 and CD80 (148–152), and we proposed a general view that PD-L1 interactions with PD-1 and CD80 could differentially regulate GVHD and GVL, as summarized in our previous review (153). In the current review, we elaborate on how PD-L1 expressed by host parenchymal tissues or expressed by donor- and host-type lympho-hematopoietic cells regulates GVHD and GVL activity mediated by the same alloreactive T cells. Parenchymal tissue expression of PD-L1 can effectively tolerize infiltrating T cells by interaction with PD-1 on activated T cells and induction of T cell anergy, exhaustion, and apoptosis, together with expansion of FoxP3^+^ Treg cells and FoxP3^-^IL-10^+^ Tr1 cells (154–156). Nonetheless, upregulation of PD-L1 by host tissues did not effectively prevent aGVHD, although it can reduce the severity of aGVHD as indicated by exacerbation of aGVHD in PD-L1^-/-^ recipients and with PD-1^-/-^ donor T cells. In addition, transgenic expression of PD-L1 by hepatocytes *via* hydrodynamic injection of PD-L1 plasmid ameliorated aGVHD with expansion of FoxP3^+^CD4^+^ Treg cells (157). The lack of effective prevention of aGVHD by host-tissue PD-L1 may result from the cytokine environment, since GVHD target tissues express elevated levels of IL-2, IFN-γ, TNF-α, GM-CSF, and IL-6 (7, 158). Neutralizing TNF-α or IL-6 did not effectively prevent aGVHD, although the severity of GVHD was reduced in murine recipients and in human HCT recipients (87–89, 91, 92). Thus, other cytokines may regulate the effects of PD-L1/PD-1 pathway.

## Host Tissue PD-L1 and Tolerogenic Anti-IL-2 mAb

**Figure 1:**
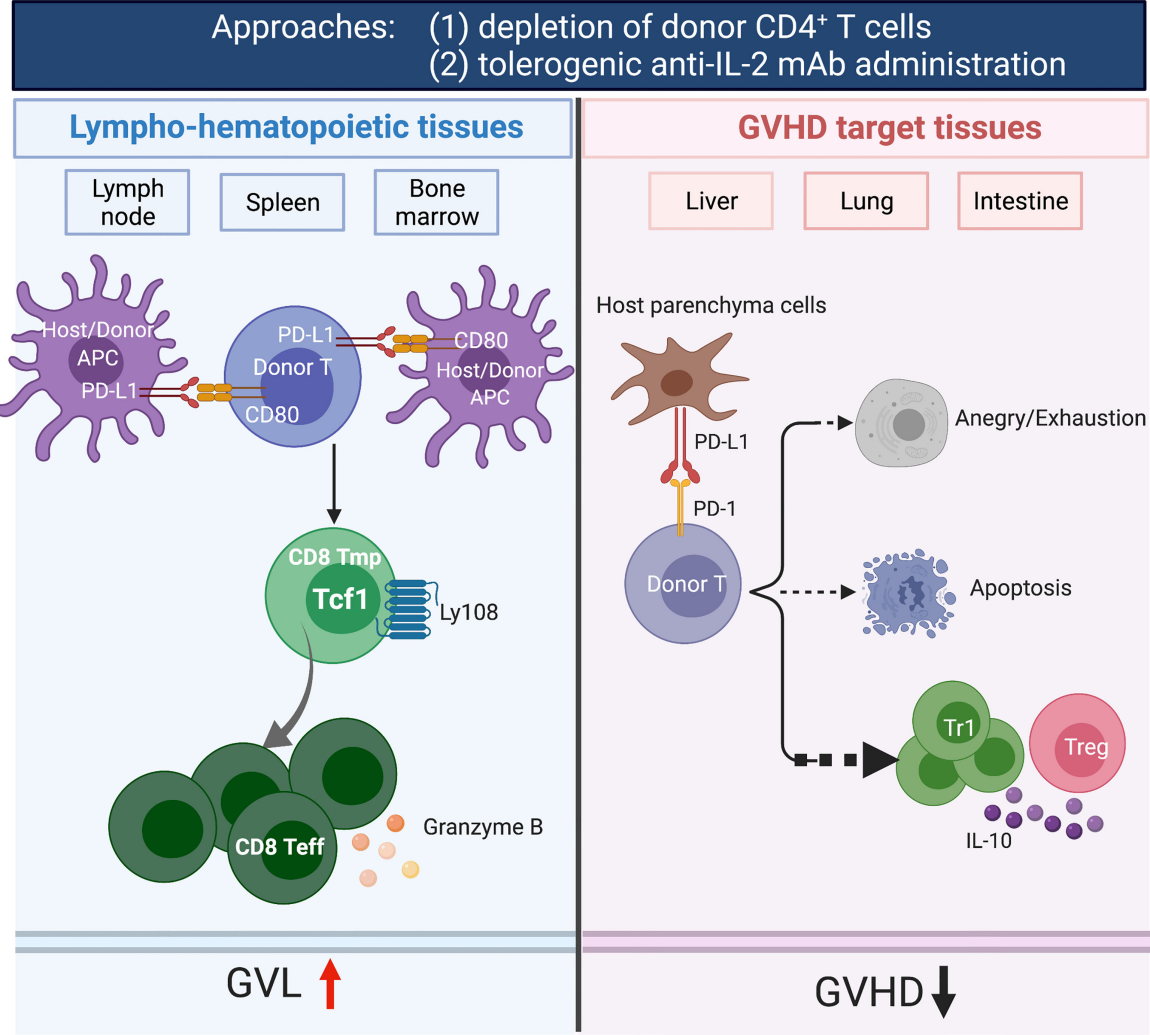
Depletion of donor CD4^+^ T cells and tolerogenic anti-IL-2 mAb (JES6-1) administration prevents acute GVHD while preserving GVL activity. The depletion of CD4^+^ T cells by anti-CD4 mAb prevented both acute and chronic GVHD while preserving GVL activity. In GVHD target tissues, depletion of CD4^+^ T cells allowed host tissue PD-L1 interaction with PD-1 expressed by donor CD8^+^ T cells to induce anergy, exhaustion and apoptosis. In lymphoid tissues, the treatment allowed PD-L1 and CD80 interactions among lymphocytes and DCs to augment expansion of CD8^+^ T cells that mediating GVL activity. Furthermore, CD4^+^ T cells help CD8^+^ T cells *via* their production of IL-2. Administration of tolerogenic anti-IL-2 mAb (JES6) expanded IL-10^+^FoxP3^-^CD4^+^ Tr1 cells in GVHD target tissues. In addition, increased expression of granzyme B and generation of TCF-1^+^CD8^+^ memory progenitors that can give rise to cytotoxic effector cells in lymphoid tissues, leading to effectively prevention of aGVHD while preserving GVL activity. Created with BioRender.com.

Sorted donor CD4^+^ T cells can cause severe GVHD by expressing FASL and producing proinflammatory cytokines (e.g., IFN-γ and TNF-α) (159, 160), while sorted donor CD8^+^ T cells prevent graft rejection and mediate GVL effects by expressing perforin/granzyme, without causing aGVHD in animal models (161, 162). IL-2 produced by CD4^+^ T cells makes CD8^+^ T cells resistant to anergy and apoptosis induced by PD-1 signaling (163). We found that administration of tolerogenic anti-IL-2 mAb (JES6) that specifically blocks IL-2 interaction with IL-2Rβ effectively prevents aGVHD while preserving strong GVL effect in a host tissue PD-L1-depdent manner. In GVHD target tissues, blockade of IL-2β signaling increased inhibition of AKT-mTOR pathway mediated by PD-L1/PD-1 signaling, upregulated T cell expression of PD-1 and Blimp-1, and expanded IL-10^+^FoxP3^-^CD4^+^ Tr1 cells (156). In lymphoid tissues, donor CD8^+^ T cells expanded and had increased expression of granzyme B and generation of TCF-1^+^CD8^+^ memory progenitors that can give rise to cytotoxic effector cells, which contribute to strong GVL activity (156). Maintenance of donor CD8^+^ T cells in lymphoid tissues may result from the lack of host-type PD-L1 expression and lack of PD-L1/PD-1 signaling. Thus, administration of tolerogenic anti-IL-2 that specifically blocks IL-2Rβ signaling may represent a novel approach for preventing aGVHD while preserving strong GVL activity through the expansion of functional CD8^+^ T cells in lymphoid organs while inducing Tcon anergy/exhaustion in GVHD target tissues (**Figure 1**). An anti-human IL-2Rβ mAb has been developed (164) but has not been evaluated in clinical trials.

Administration of tolerogenic anti-IL-2 mAb that blocks IL-2 interaction with IL-2Rβ did not prevent cGVHD (156). Although the treatment was very effective at prevention of gut aGVHD, the recipients developed cGVHD with body weight loss (156). The lack of protection of thymus may result from autoreactive PD-1^hi^IFN-γ^+^IL-10^+^CD4^+^ T cell interactions with B cells. Our previous report showed that autoreactive IFN-γ^+^IL-10^+^CD4^+^ T cells can activate B cells to produce autoantibodies (165), and donor-type tissue-resident PD-1^hi^CD4^+^ T helper cells interact with B cells in the GVHD target tissues to produce autoantibodies that mediate thymus damage and cutaneous GVHD (166). Those PD-1^hi^ T helper cells were derived from naïve CD4^+^ T cells in the graft (166), depletion of naïve T cells in the graft was recently reported to effectively prevent cGVHD (140).

## Host Tissue PD-L1 and Depleting Anti-CD4 mAb

aGVHD is mediated by donor CD4^+^ and CD8^+^ T cells in the graft, and cGVHD is mediated mainly by CD4^+^ T cells from the graft and from T cells that are *de novo* generated from progenitors in the GVHD-damaged thymus (167, 168). With a murine model that reflects characteristic features of acute and chronic GVHD, we showed that sorted CD4^+^ T cells induce both acute and chronic GVHD. Sorted CD8^+^ T cells did not induce aGVHD but did induce cGVHD that depended on *de novo* generated CD4^+^ T cells (169). In follow-up studies, we administered a depleting anti-CD4 mAb weekly for 4 weeks after HCT to deplete donor CD4^+^ T cells derived from the graft and from *de novo*-regeneration early after HCT (163). Notably, the administration of anti-CD4 mAb did not affect bone marrow reconstitution and allowed full recovery of donor CD4^+^ T cells on day 100 after HCT (163). The depletion of CD4^+^ T cells by anti-CD4 mAb prevented both acute and chronic GVHD while preserving strong GVL activity in murine and humanized xeno-GVHD models (163). In GVHD target tissues, depletion of CD4^+^ T cells allowed host tissue PD-L1 interaction with PD-1 expressed by donor CD8^+^ T cells to induce anergy, exhaustion and apoptosis. In lymphoid tissues, however, the treatment allowed PD-L1/CD80 interactions to augment expansion of CD8^+^ T cells early after HCT, which contributed to strong GVL activity (163). CD4^+^ T cells help CD8^+^ T cells *via* their production of IL-2 that prevents T cell tolerance induced by PD-1 signaling (163). Deletion of CD4^+^ T cells not only removed the IL-2 effect on donor CD8^+^ T cells and augmented infiltrating CD8^+^ T tolerance but might also prevented formation of tissue resident CD4^+^ T cell helpers for B cells that mediate cGVHD. Administration of depleting anti-CD4 mAb may also deplete the pre-existing host-type tissue-resident CD4^+^ T cells in GVHD target tissues that augment induction of aGVHD (66). Therefore, administration of depleting anti-CD4 mAb early after HCT may represent one of the most effective approaches to prevent acute and chronic GVHD while preserving strong GVL activity (**Figure 1**).

## Donor- and Host-Type Hematopoietic Cell Expression of PD-L1 Differentially Regulate Alloreactive T Cell Expansion and GVL Activity

PD-L1 is induced and constantly expressed by parenchymal cells in inflamed GVHD target tissues (163, 170). Host-type hematopoietic cells in the lympho-hematopoietic tissues are rapidly eliminated and replaced by donor-type cells early after HCT (171). Donor-type cells in lymphoid tissues expressed higher levels of PD-L1 and CD80 but lower levels of PD-1 as compared to those in the GVHD target tissues (163). Thus, PD-L1 interaction with CD80 in the lymphoid tissues is likely dominant in lymphoid tissues, while PD-L1 interaction with PD-1 is dominant in GVHD target tissues, although both interactions exist in the two compartments. Accordingly, PD-L1 interactions with PD-1 and CD80 differentially regulate GVHD and GVL activity (163).

Blazar’s group showed that while host-tissue PD-L1 ameliorated aGVHD, donor cell PD-L1 augmented T cell expansion and aGVHD (172). Since PD-L1 interaction with PD-1 always inhibits T cell expansion (173), the role of donor T cell PD-L1 on augmenting T cell expansion and GVHD must be through PD-L1 interaction with CD80 or other ligands. Consistently, we observed that PD-L1 or CD80 deficiency in donor T cells and specific blockade of PD-L1 interactions with CD80 by anti-PD-L1 mAb (43H12) given on day 0 before T cell activation *in vivo* reduced CD8^+^ T expansion (163). Blockade of PD-L1 interaction with CD80 after T cell activation on day 5 after HCT, however, augmented donor CD8^+^ T expansion (174). Although we assumed that PD-L1 interaction with CD80 occurred in trans (151, 157, 175), recent publications showed that PD-L1 interactions with CD80 do occur in cis and that PD-L1/CD80 interactions in cis on APCs reduced PD-L1 interaction with PD-1 and reduced CD80 interaction in trans with CTLA-4 on T cells, thereby augmenting CD8^+^ T cell expansion (176, 177). Our studies, however, showed that *in vivo* 43H12 blockade augmented expansion of CD44^+^CD62L^-^CD8^+^ memory/effector T cells in tumor draining lymph nodes (178). This effect is opposite from blocking cis PD-L1/CD80 interactions, suggesting that trans PD-L1/CD80 interaction occurs *in vivo*. Furthermore, these findings correlate with the observation that blockade at day 5 accelerated the expansion of donor CD8^+^ T cells in allogeneic recipients (174). Naïve T cell expressed low levels of PD-L1 and CD80, while activated T cells and APC express high levels of PD-L1 and CD80 (151). Thus, administration of 43H12 on day 0 predominantly blocks PD-L1/CD80 cis interactions on host APC (176, 177), while administration of 43H12 on day 5 primarily blocks trans PD-L1/CD80 interactions (153, 157).

Taken together, our studies suggest that when donor T cells interact with host APCs in lymphoid tissues early after HCT, the interaction of PD-L1 on donor T cells with CD80 on host-APCs augments donor T cell expansion. After donor T cells are fully activated and host APCs in the lymphoid tissues are eliminated, donor T cells interact with donor APCs, and the PD-L1/CD80 between donor T cells and APCs augment the tolerance effect of PD-1/PD-L1 interactions. This hypothesis is supported by our observation that blockade of PD-L1/CD80 interaction between activated donor CD4^+^ T cells and APCs in allogeneic recipients augmented the expansion of the CD4^+^ T cells, and the effect occurred only in WT donor T cells but not in PD-1^-/-^ donor T cells (157).

Administration of anti-PD-1 or anti-PD-L1 mAb to the patients who with tumor relapsed after allo-HCT induced lethal aGVHD (179–182). Since blockade of PD-L1/CD80 interaction reduced but did not completely remove the inhibitory effect of PD-L1/PD-1 interactions, we expect that administration of antibodies to specifically block PD-L1/CD80 interaction will augment GVL effect in lymphoid tissues while maintaining the protective effect of PD-L1/PD-1 interactions in GVHD target tissues. Therefore, blockade of PD-L1/CD80 in patients with relapse could augment GVL activity with little GVHD.

## Sequential Administration of Tolerogenic Anti-IL-2 and JAK Inhibitor

JAKs are intracellular signaling components that function as downstream signal mediators for many cytokines (183). The JAK family contains four members. Among these, JAK1, JAK2, and JAK3 may be important for the development of GVHD (184–188). JAKs regulate the function of immune cells that mediate GVHD, including APCs (189), T cells (190), and B cells (191). Thus, numerous studies have been conducted to investigate the role of JAKs inhibitors in regulating GVHD in preclinical models. John F. DiPersio’s group evaluated the effect of JAK1/JAK2 inhibitors in an MHC-mismatched murine model and showed that JAK1/JAK2 inhibitors inhibited IFNR and IL-6R signaling, which inhibited migration of alloreactive T cells to GVHD target organs by decreasing expression of CXCR3. JAK1/JAK2 inhibition also expanded Tregs, and the two effects effectively prevented GVHD (184, 192). Similarly, inhibition of JAK1/JAK3 inhibition also reduced aGVHD and enhanced survival (187). While significant evidence supports the role of multi-kinase inhibitors that target more than one JAK protein, selective JAK1, JAK2 or JAK3 inhibition is also effective in many GVHD models (188, 193, 194). The impact of JAK inhibitors on GVL activity, however, is variable. For example, Baricitinib (JAK1/JAK2 inhibitor) enhanced GVL effects by downregulating PD-L1 expression in tumors (192). While Ruxolitinib (JAK1/JAK2 inhibitor) impaired murine CTL activity against tumor cells *in vitro*, neither pacritinib (JAK2 inhibitor) nor ruxolitinib interfered with the GVL effect *in vivo* in MHC-mismatched murine models (186, 194). In xenograft models, however, ruxolitinib significantly impaired antitumor activity against U937 cells, while only pacritinib preserved CTL function (194). The success of many clinical studies evaluating the efficacy of JAK inhibitors for treatment of both SR-aGVHD and SR-cGVHD (185, 195–200), have prompted interest in testing JAK inhibitors for prevention of GVHD (201, 202). Since our previous study showed tolerogenic anti-IL-2 mAb effective prevent aGVHD and maintain GVL activity, but did not prevent cGVHD (156). It would also be of interest to test whether sequential administration of tolerogenic anti-IL-2 mAb and JAK inhibitors will effectively prevent both aGVHD and cGVHD while preserving GVL activity.

## Summary

**Figure 2:**
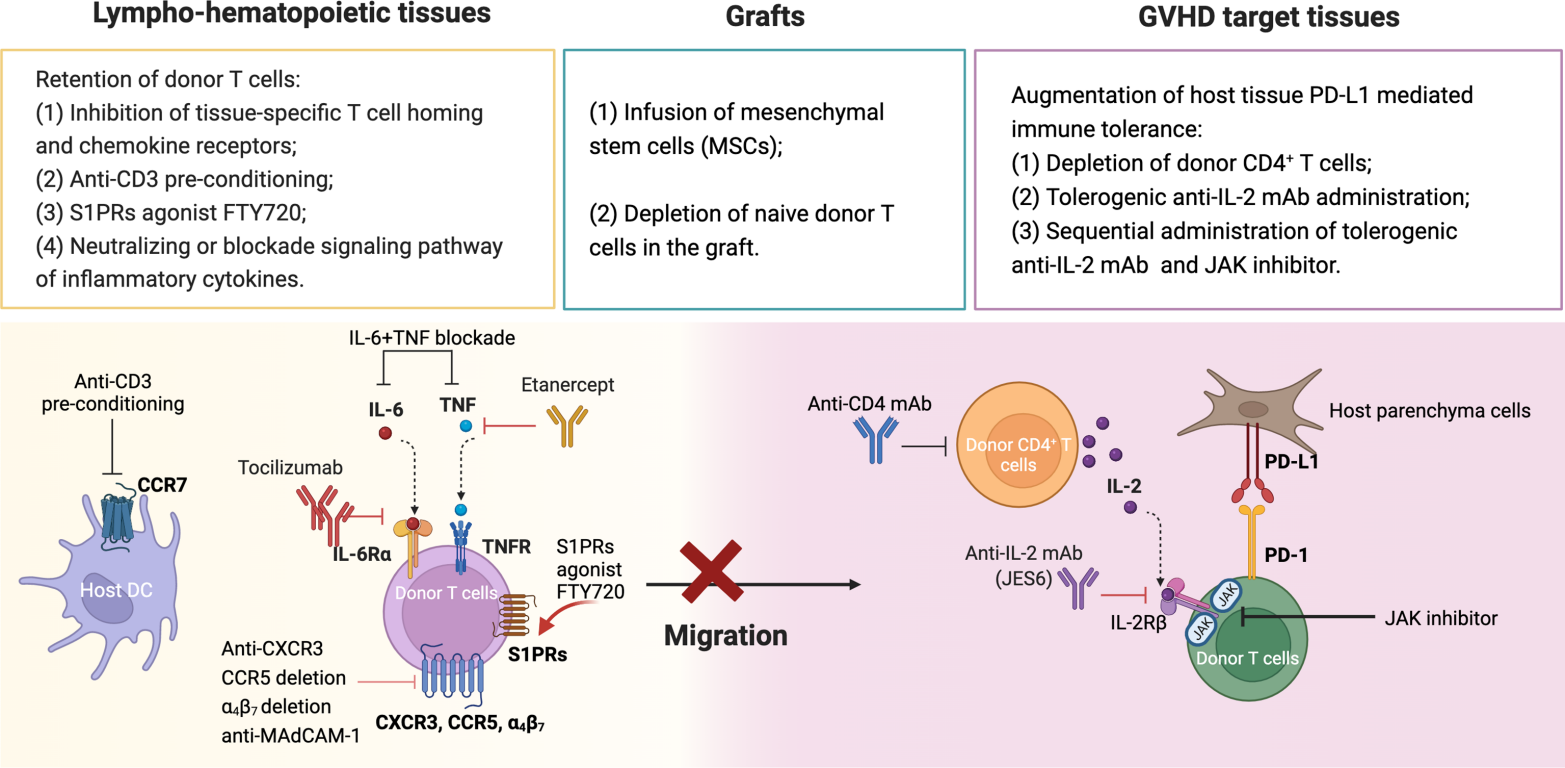
Summary of approaches for prevention of GVHD while preserving GVL activity. (1) Retention of donor T cells in lympho-hematopoietic tissue. (2) Depletion of naïve donor T cells and infusion of mesenchymal stem cells. (3) Augmentation of tissue PD-L1 mediated immune tolerance. Created with BioRender.com.

The cellular interactions that lead to GVHD occur in the skin, liver, gut and lung, while those that lead to GVL activity occur in lympho-hematopoietic tissues (12, 61). We summarized the approaches that GVHD could be prevented while preserving GVL activity in **Figure 2**. First, inhibiting alloreactive T migration and expansion in GVHD target tissues while allowing full activation and expansion of alloreactive T cells in lympho-hematopoietic tissues. Approaches that specifically prevent alloreactive T cell infiltration into GVHD target tissues include the following (**Table 1**): 1) targeting chemokine or chemokine receptors, such as CCR5, CXCR3, MAdCAM-1; 2) anti-CD3-preconditioning that depletes host DCs that imprint alloreactive T cell tissue tropism; 3) FTY720 that prevents alloreactive T cell egress from lymphoid tissues; and 4) neutralizing or blockade signaling pathways of inflammatory cytokines such as TNF-α and IL-6. Some of these approaches (i.e., FTY720, anti-TNF-α and anti-IL-6R) have been tested in the clinic, but the effect was minimal (94–96), while some (i.e., anti-CD3-preconditioning, anti-CCR5) have not been tested. Depletion of naïve T cells effectively prevents cGVHD and severe aGVHD. Evidence suggests that the high incidence of mild aGVHD in these patients helps to decrease the risk of relapse without increasing the risk of non-relapse mortality. Other promising approaches are to augment parenchymal tissue protective mechanisms mediated by PD-L1 interaction with PD-1 and CD80, including 1) administration of depleting anti-CD4 mAb that allows parenchymal tissue PD-L1/PD-1 interaction to tolerize infiltrating donor CD8^+^ T cells while allowing lymphoid tissue PD-L1 interaction with CD80 to augment expansion of donor CD8^+^ T cells that mediate GVL activity (163) (2); administration of tolerogenic anti-IL-2 mAb early after HCT that prevents aGVHD while preserving strong GVL activity (156), potentially in combination with JAK inhibitors to prevent cGVHD.

**Table 1:** Approaches that prevent alloreactive T cell infiltration into GVHD target tissues.

Strategies	Preclinical studies	Clinical applications	References
Targeting chemokine or chemokine receptors	anti-CXCR3, CXCR3 transfected Tregs, CCR5 deletion, α_4_β_7_ deletion, anti-MAdCAM-1	CCR5 deletion mutation in both donor and recipient decreased GVHD.	(26–28, 30–35, 38, 41–43)
Anti-CD3 preconditioning	Depletes host CCR7^+^ DCs in the draining lymph nodes and markedly reduces alloreactive T cell tissue tropism for gut, liver, lung and skin.	N/A	(54, 55)
S1PRs agonist FTY720	Retains alloreactive T cells in the lymphoid tissues and prevents T cell infiltration of GVHD target tissues.	A 66-year-old patient with severe CNS GVHD treated successfully.	(56–62)
Neutralizing or blockade signaling pathway of inflammatory cytokines	Anti-TNF ameliorates GVHD while preserving GVL effects in experimental murine models. Anti-IL-6R inhibits Th1 and Th17 cells while expanding Tregs, thus preventing GVHD in murine models. Combined blockade of both TNF and IL-6R prevents GVHD but does not impair GVL effects.	Anti-IL-6 for GVHD prophylaxis had no improvement in long term-survival. Addition of etanercept (TNF inhibitor) to a standard GVHD prophylaxis regimen delayed development of aGVHD but had no favorable impact on cGVHD.	(84–93)

## Author Contributions

QS wrote the review manuscript; UN edited the manuscript; RN, PM, and DZ critically reviewed and edited the manuscript. All authors contributed to the article and approved the submitted version.

## Funding

This work was supported by National Institutes of Health Grant R01 CA228465 (to DZ) and National Natural Science Foundation of China (grant no. 82100226) to QS.

## Conflict of Interest

The authors declare that the research was conducted in the absence of any commercial or financial relationships that could be construed as a potential conflict of interest.

## Publisher’s Note

All claims expressed in this article are solely those of the authors and do not necessarily represent those of their affiliated organizations, or those of the publisher, the editors and the reviewers. Any product that may be evaluated in this article, or claim that may be made by its manufacturer, is not guaranteed or endorsed by the publisher.
